# Supplementary material for: PHAGE Study: Effects of Supplemental Bacteriophage Intake on Inflammation and Gut Microbiota in Healthy Adults
Source: Nutrients. 2019 Mar 20;11(3):666. doi: 10.3390/nu11030666 (PMC6471193; doi:10.3390/nu11030666)
Supplement: Supplementary file 1 [file nutrients-11-00666-s001.pdf]

Supplementary Materials

Table S1. Microbial alpha diversity parameters.

	CHAO1	Shannon	Simpson
**Treatment (t=0d)**	38.1 (±4.83)	2.48 (±0.21)	0.86 (±0.04)
**Treatment (t=28d)**	37.8 (±4.92)	2.45 (±0.17)	0.86 (±0.04)
**Placebo (t=0d)**	37.4 (±5.05)	2.50 (±0.21)	0.87 (±0.04)
**Placebo (t=28d)**	37.8 (±6.05)	2.45 (±0.28)	0.85 (±0.06)

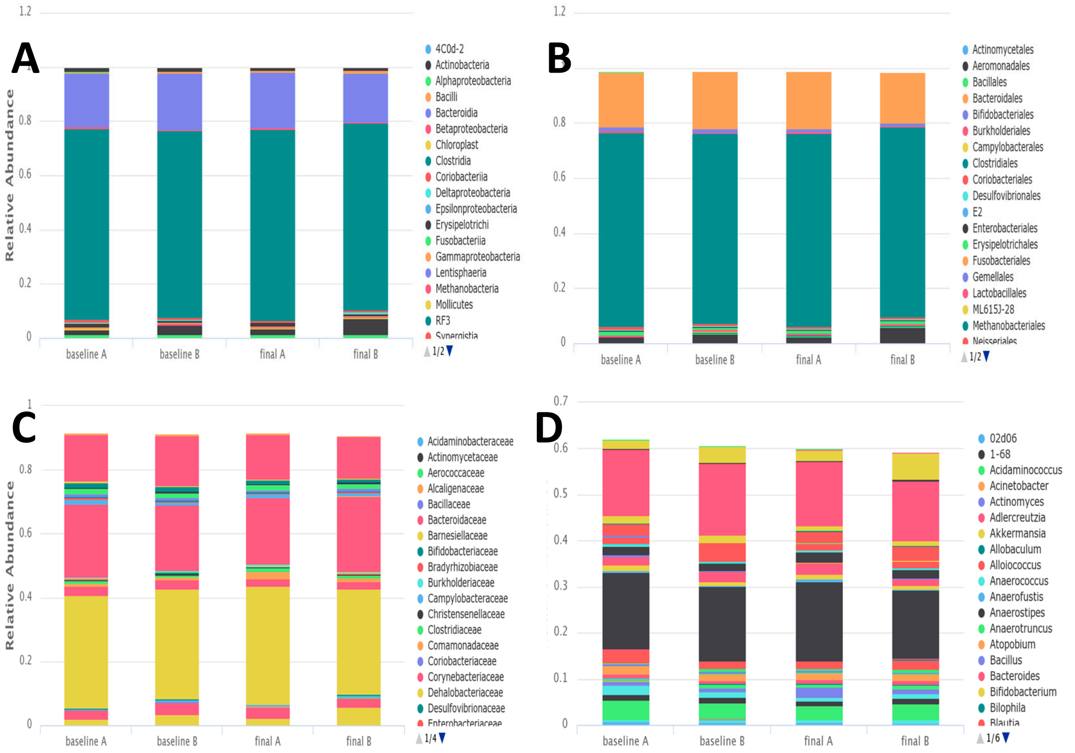

Figure S1. Bar plots showing relative abundance of taxa at (A) class, (B) order, (C) family, and (D) genus levels. Unclassified taxa were removed from the analyses. Columns marked A are treatment and B are placebo (baseline, t=0; final, t=28).

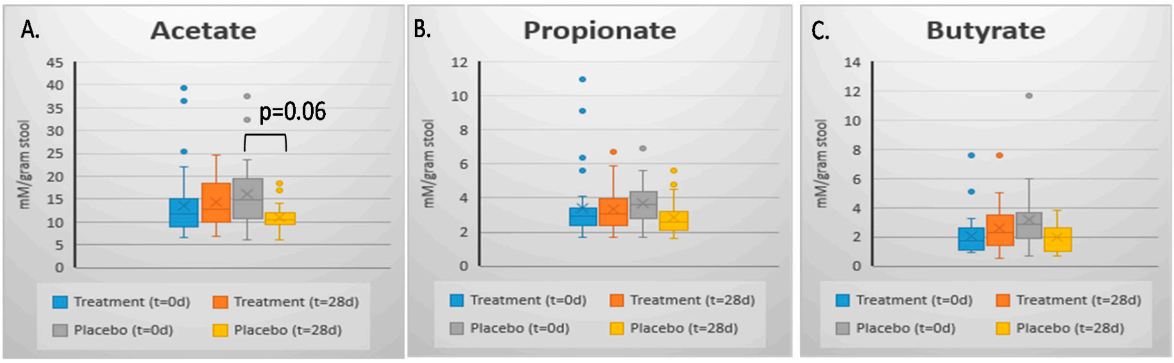

Figure S2. Fecal short chain fatty acid levels across treatments and time.

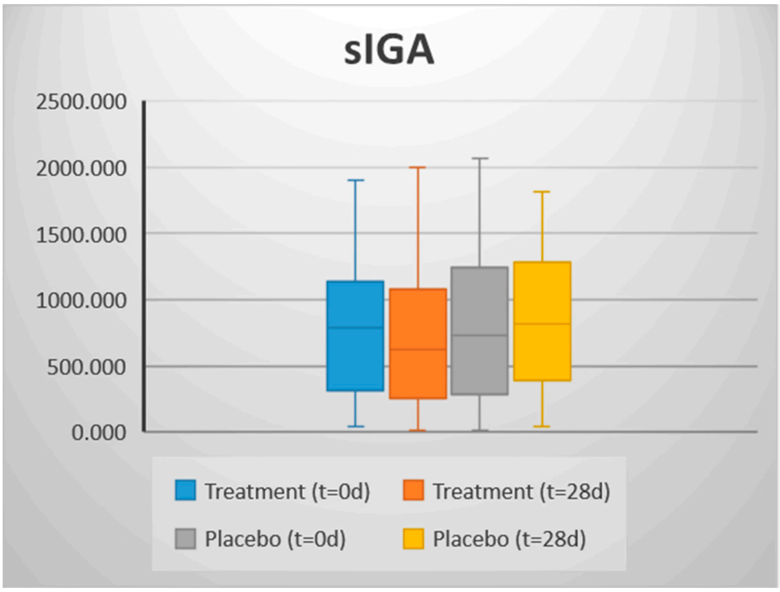

Figure S3. Fecal sIGA levels.
